# Dysphagia as an Initial Manifestation of Eosinophilic Esophagitis

**DOI:** 10.7759/cureus.7898

**Published:** 2020-04-30

**Authors:** Keerti Deepika, Ramesh Adhikari, Sreedhar Adapa, Srikanth Naramala, Venu Madhav Konala

**Affiliations:** 1 Pediatrics/Translational Research, Thomas Jefferson University, Philadelphia, USA; 2 Hospital Medicine, Franciscan Health Hospital, Lafayette, USA; 3 Geriatrics, Brown University, Providence, USA; 4 Nephrology, Kaweah Delta Medical Center, Visalia, USA; 5 Rheumatology, Adventist Medical Center, Hanford, USA; 6 Hematology and Oncology, Ashland Bellefonte Cancer Center, Ashland, USA; 7 Hematology and Oncology, King's Daughters Medical Center, Ashland, USA

**Keywords:** eosinophilic esophagitis, esophageal stenosis, intra-epithelial eosinophilia, allergic rhinitis, sublingual immunotherapy

## Abstract

Eosinophilic esophagitis (EoE) is a clinicopathological condition characterized clinically by symptoms of esophageal dysfunction, with typical endoscopic findings and intra-epithelial eosinophilia on biopsy. This case report focuses on the historical aspect of EoE, clinical manifestations, and correlation with immune disorders, medical management, and interventional management of EoE. We present a 20-year-old patient presenting with tightness in throat and odynophagia after the ingestion of certain foods. These symptoms resolve in two or three hours. Endoscopic examination of the upper gastrointestinal tract visualizes esophageal stenosis, and histological examination of the biopsy specimen reveals increased eosinophils in the esophageal mucosa. The patient was treated with fluticasone inhaler and has shown improvement in symptoms. EoE is a chronic esophageal disorder that is increasing in incidence and prevalence in both pediatric and adult age groups. This case report accentuates the complications of EoE, and delays in diagnosis lead to strictures, and fibro-stenotic disease and punctual recognition can govern the course of the disease.

## Introduction

Eosinophilic esophagitis (EoE) is a clinicopathologic entity distinguished clinically by a pattern of symptoms related to esophageal dysfunction and histologically by intraepithelial eosinophilia on biopsy [1, 2]. It is a chronic, allergic inflammatory disease of the esophagus that is being discerned with expanding frequency and is now pondered a vital cause of gastrointestinal illness [3]. EoE predominantly presents with dysphagia and esophageal food impaction, along with persistent heartburn and regurgitation in adults [4]. Symptoms frequently mimic gastroesophageal reflux disease (GERD), but both of these diseases are distinct in gene expression and signature, response to therapy, genetic risk, an association with allergies, and histopathology [5]. The diagnosis of EoE requires a histological finding of greater than 15 intraepithelial eosinophils in at least one high power field (HPF) in esophageal mucosa [6]. Initially, the reports were predominantly from the pediatric population, where children suffering from allergy presented with severe GERD-like symptoms, which are refractory to medical or surgical therapy. They also had infiltration of the esophagus with eosinophils and responded to a hypoallergic diet [7]. However, as more gastroenterologists biopsied the patients with dysphagia, the more frequent the diagnosis was found in adults. We present a case of eosinophilic esophagitis in a 20-year-old male with symptoms of tightness and swelling in his throat and odynophagia.

## Case presentation

A 20-year-old Caucasian male presents with difficulty swallowing for the last three years. He started to develop tightness in throat and odynophagia after ingesting foods like banana and individual salads. It takes one or two hours for the symptoms to resolve. It is not associated with dyspnea, cough, nausea, or wheezing with food ingestion. Otolaryngology consultation advised initial imaging with a barium swallow, which revealed concentric rings in the esophagus.

Past medical history comprises of seasonal allergic rhinitis since childhood with frequent episodes of itchy watery eyes, sneezing, nasal congestion, clear rhinorrhea, sinus pressure, headache, and postnasal drip during early spring and fall season. Over the counter, anti-histaminic medications provided symptomatic relief during allergic episodes. He also had a history of asthma, which was diagnosed at age 3 but was resolved by age 6. He has no known food or drug allergies. He is a non-smoker, non-alcoholic, and denies any drug abuse. The patient has no history of sublingual immunotherapy (SLIT) for allergy. A review of other systems was non-contributory, and physical examination was unremarkable.

**Figure 1:**
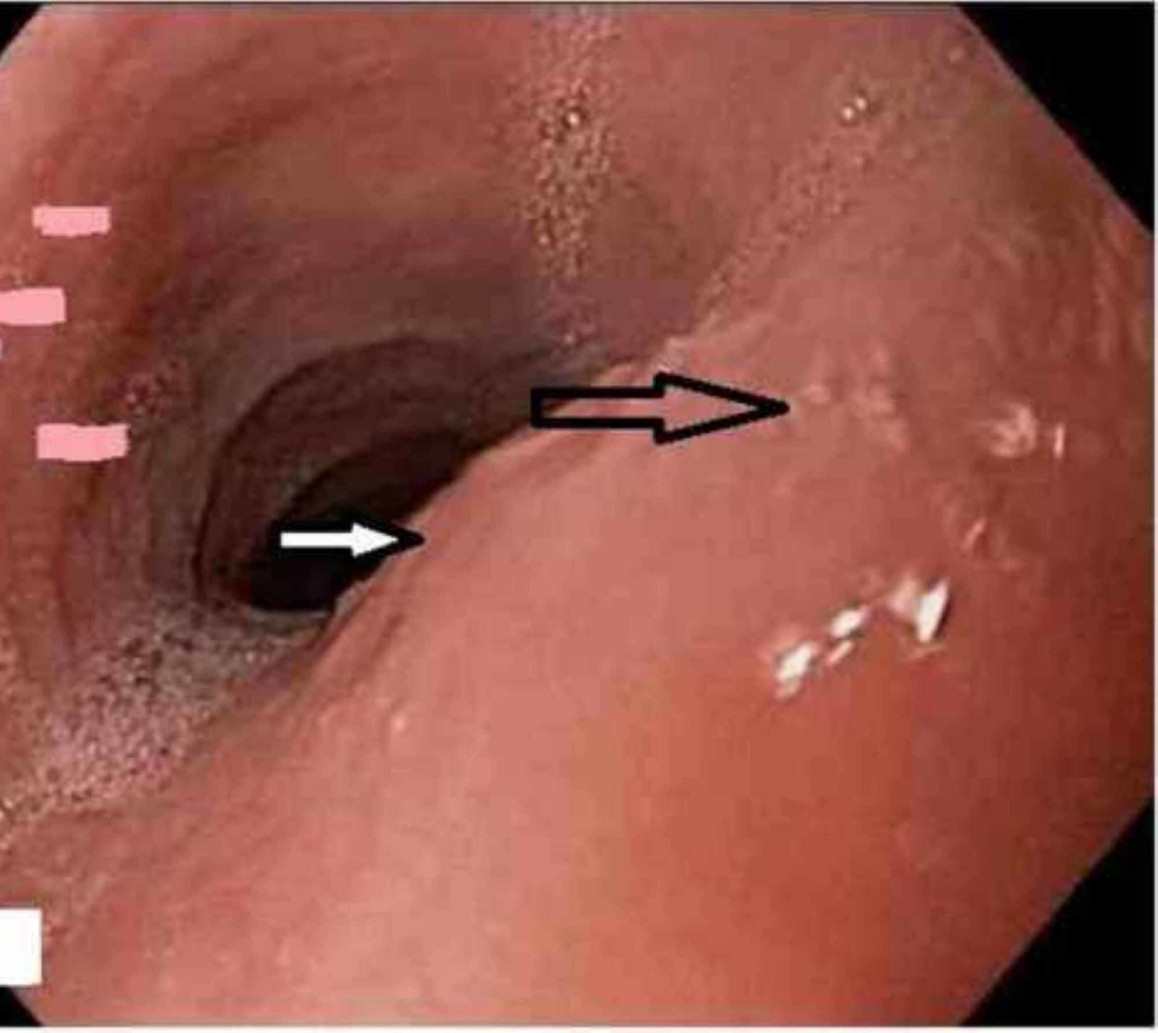
Diagnostic endoscopy showing esophageal stenosis less than 1 cm in length and multiple rings (pointed out with arrows).

**Figure 2:**
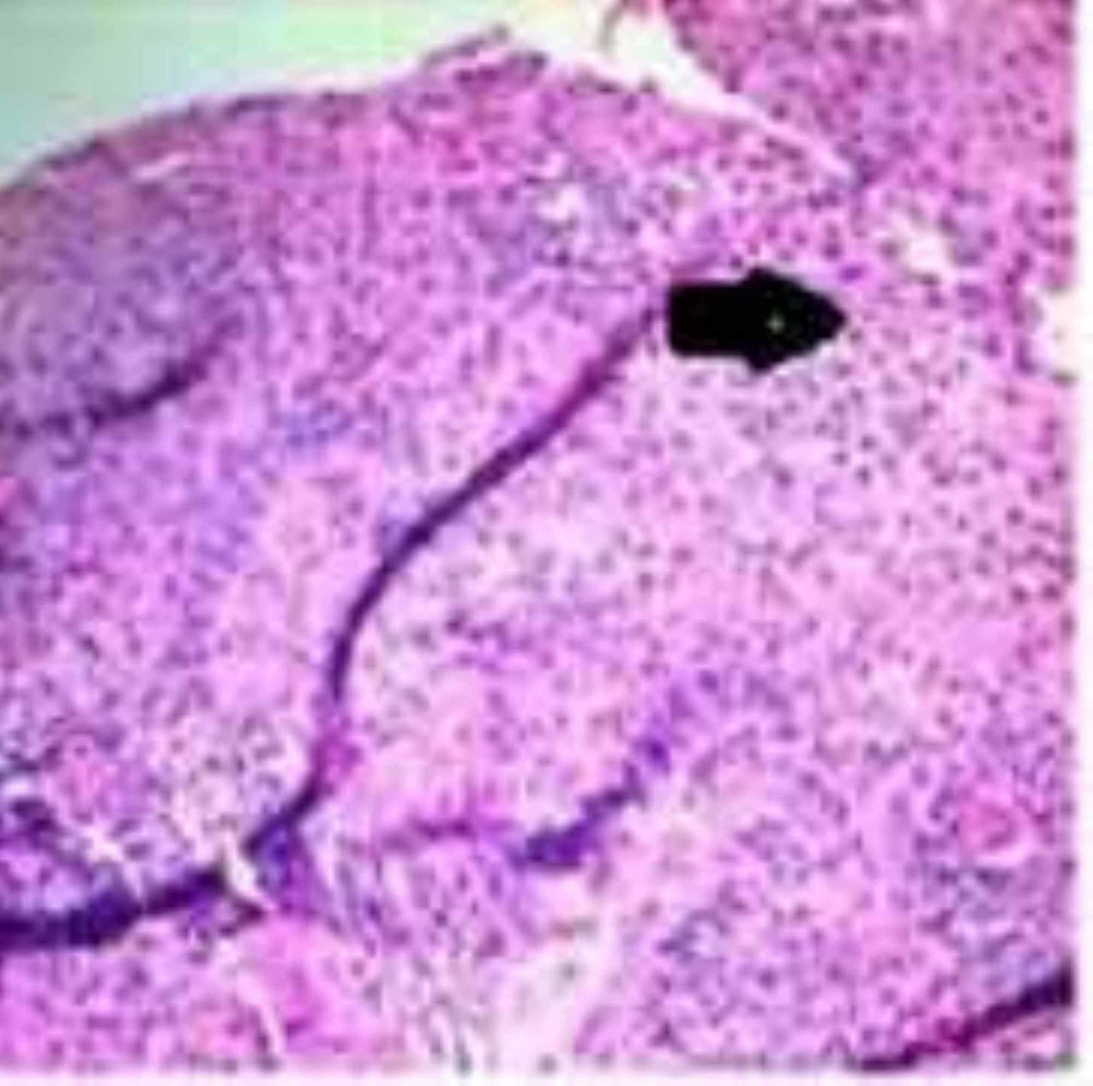
Histopathological examination showing marked increase in intraepithelial cells per high power field.

Diagnostic endoscopy visualized benign-appearing esophageal stenosis measuring less than 1 cm in length and 1 cm in diameter, and it was found 25 cm from incisors and is non-traversable. Multiple rings were found distally (Figure 1). Histological examination of the biopsy specimen revealed elongation of submucosal papillae in the squamous mucosal surface, extensive basal cell hyperplasia and abundant intraepithelial eosinophils (25 eosinophils/high power microscopic field) with occasional eosinophilic microabscesses mostly prominent in the superficial aspect of the mucosa which would favor a diagnosis of EoE (Figure 2).

**Figure 3:**
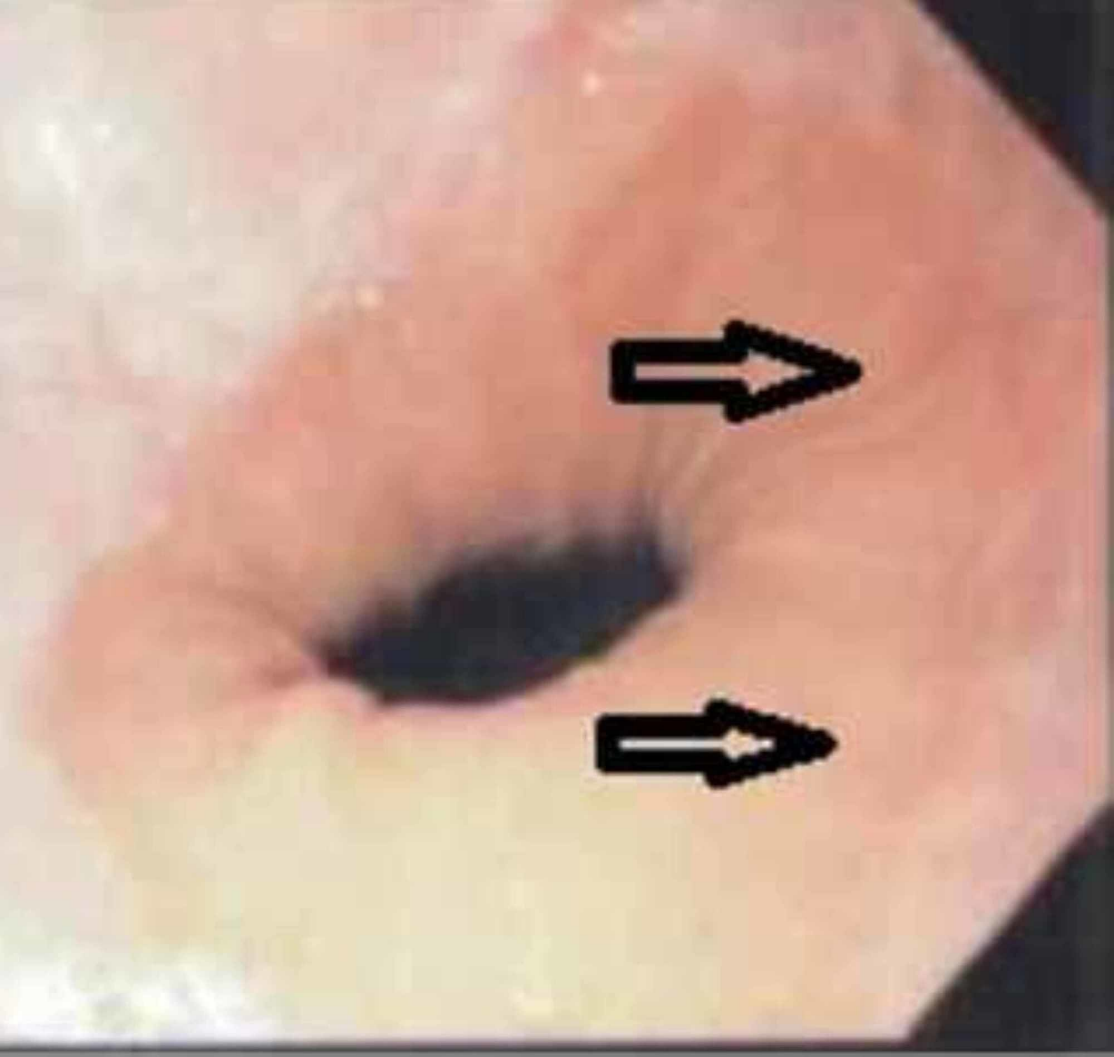
Diagnostic endoscopy showing significant improvement compared to Figure 1.

The patient was treated with fluticasone 250 micrograms as a multiple-dose inhaler, with four puffs swallowed twice a day for eight weeks. His symptoms improved gradually, and an upper endoscopy performed after three months showed improvement in endoscopic appearance (Figure 3). Histopathological examination of the biopsy specimens also revealed a marked decrease in the number of intra-epithelial eosinophils per high power field in the esophageal mucosa. The patient remained in remission at the 12-month follow-up.

## Discussion

EoE was first elucidated in 1978, where an isolated case of severe achalasia in a patient with significant smooth muscle hypertrophy and eosinophilic infiltration of esophageal mucosa predisposed to esophageal motor disorder [8]. In 1989 esophageal asthma - episodic dysphagia with eosinophilic infiltrates - was described after a study was conducted in two groups with GERD and dysphagia where dense esophageal eosinophilia of more than 20 eosinophils per high power field was observed in patients with dysphagia and this was termed as esophageal eosinophilia with dysphagia, which is a distinct clinicopathological syndrome [9,10]. The best corroboration for this comes from investigators in Switzerland in 1989, who created a database of EoE patients [11]. The patient population and health care providers did not change conspicuously over 15 years, however, they noted an increase in the incidence of EoE from 1989 to 2004 by three-fold [11]. Since 1990, EoE has transformed from a rare reportable condition to a disease that is frequently encountered in the clinic and endoscopy suite. It is a significant cause of upper gastrointestinal morbidity and increasing health care costs. EoE appears to have male predominance in children as well as adults. Although the pathogenesis of EoE is not fully understood, it is concomitant to allergic disorders like food hypersensitivity.

The incidence and prevalence of EoE are increasing at rates that outpace increased recognition but is arduous to estimate due to the paucity of resources for work up in certain countries. Presently, it seems that as more gastroenterologists biopsied the esophagus of patients with dysphagia, it increased the rate of detection and frequency of diagnosis in adults.

Increased number of eosinophils in the gastrointestinal tract has been documented in a variety of diseases including EoE, Crohn's disease, Eosinophilic gastroenteritis, GERD, infections, hypereosinophilic syndrome, scleroderma, iatrogenic-induced states such as caustic injury and immunosuppression following solid organ transplantation [5]. Emerging evidence in the pathologic mechanism of EoE suggests that it is mediated by type 2T helper cell immune response like many other allergic diseases [12]. More than 70% of the patients with EoE have a history of allergic conditions like asthma, allergic rhinitis, eczema, and hypersensitivity to certain foods [12].

Symptoms of GERD and EoE generally imbricate with each other and can be distinguished by various factors. Initial studies propounded mucosal injury caused by acid reflux may allow swallowed allergens to penetrate esophageal mucosa, causing eosinophilia. Therefore, EoE is generally distinguished from GERD by persistent esophageal eosinophilia despite adequate acid neutralization therapy before endoscopy [5]. Histological examinations of GERD and EoE share various degrees of eosinophilia. In GERD, eosinophilic infiltration is less dense that is less than 5 per high power field and mostly found in the distal end of the esophagus. In contrast, in EoE, it is denser, with more than 20 per high power field, and the eosinophilic infiltration is found throughout the esophagus, including the proximal, middle and distal end [10, 13].

In EoE, eosinophils, along with other inflammatory cells, including T lymphocytes, mast cells, and dendritic cells, are observed [5]. A significant finding is the activation of esophageal eosinophils in patients with EoE which implies eosinophils as a marker in the diagnosis of EoE as well as a part of the pathophysiology and in certain cases, the esophageal fibrosis correlates with the extent of eosinophil activation rather than the density of eosinophils per high power field. Eotaxin and Interleukin 5 are observed to be the primary recruiters of eosinophils to the esophagus [5].

Also, in patients with EoE, the total serum IgE is observed to be increased, and peripheral eosinophilia is uncommon, and in many patients history of atopic allergy is common and is mostly related to allergic disorders especially food antigen sensitization. Food sensitization is repeatedly observed in patients with EoE. Food hypersensitivity is characterized into IgE mediated, and Non-IgE mediated, where IgE mediated causes an immediate reaction and detected by skin prick and food-specific IgE levels in the serum. In contrast, non-IgE mediated reasons delayed response and detected by atopy tests. EoE is considered as a mixed reaction disorder, and the combination of both tests is competent to identify the causative foods.

Diagnostic studies

A diagnostic endoscopic examination may reveal rings, corrugation, linear furrows, plaques, whitish exudates, and friable mucosa [13, 14]. Biopsy specimens should be taken from proximal and distal esophagus even if mucosa appears normal in endoscopy in patients with unexplained solid food dysphagia [13, 14]. Recent studies specify that mid-esophageal specimens should also be taken. Less commonly, strictures can be found on barium radiography, and decreased caliber of the esophageal lumen can be observed [14]. Esophageal manometry is not recommended as a routine test as it is of limited diagnostic value.

Management of EoE

Dietary management of EoE is the most effective and first-line non-pharmacological treatment of EoE. Since most of the patients with EoE present with food hypersensitivity, recognition, and avoidance of certain foods that cause the reaction is the first approach. The medical management of EoE is summarized in Table 1 [13, 15].

**Table 1:** Medical management of eosinophilic esophagitis.

AGENT	ROUTE OF ADMINISTRATION	EFFECTS	ADVERSE REACTIONS
Proton Pump Inhibitors	Oral	1) Decreased acid secretion, 2) Anti-inflammatory	Headache, nausea, diarrhea
Topical Glucocorticoids	Oral	1) Decreased eosinophil counts	Abdominal pain, oral thrush, hoarseness of voice
Fluticasone propionate	Metered-dose inhaler without a spacer	1) Relief of dysphagia, 2) Decreased inflammation of the esophagus	Oral thrush, cataracts, adrenal suppression and relapse
Budesonide	Nebulizer	1) Relief of dysphagia, 2) Decreased inflammation of the esophagus, 3) Decreased eosinophil counts.	Oral thrush and relapse
Ciclesonide	Oral	Decreased eosinophil counts in the proximal and distal esophagus as shown in histopathology.	Unpleasant taste, dryness, and burning sensation in the mouth.

Interventional management

Esophageal dilation is suggested in patients with strictures as esophageal fibro-stenosis is commonly seen in severe cases of EoE and patients where conservative management has failed. It helps in relieving dysphagia but does not affect underlying inflammation. The risk of esophageal perforation is high during the procedure, and rigid endoscopy should be avoided [15, 16].

Other treatments like monoclonal antibodies against IL-13, dupilumab, mepolizumab, reslizumab, anti-IgE antibody, anti-TNF therapy, prostaglandin D2 receptor antagonist, cromolyn, montelukast and purine analogs are being studied in the treatment of EoE [15].

## Conclusions

In conclusion, EoE is a chronic esophageal disorder that is increasing in incidence and prevalence in both pediatric and adult age groups and needs maximal heed from clinicians as delay in diagnosis can lead to complications like strictures and fibro-stenosis. Clinicians should have a high suspicion in patients with atopic symptoms presenting with dysphagia and esophageal food impaction. This case report accentuates the complications of EoE, and delays in diagnosis lead to strictures, and fibro-stenotic disease and punctual recognition can govern the course of the disease.
